# Information vs situation: balancing transparency and autonomy for trustworthy autonomous vehicles

**DOI:** 10.3389/frobt.2025.1657857

**Published:** 2025-09-22

**Authors:** Ana Tanevska, Katie Winkle, Ginevra Castellano

**Affiliations:** Department of Information Technology, Uppsala University, Uppsala, Sweden

**Keywords:** trustworthy HRI, ethical HRI, participatory design, autonomous vehicles, human-AV interaction, user-centered design, autonomy, transparency

## Abstract

With the rapid advancement of autonomous vehicle (AV) technology, AVs move beyond their initial purpose of only providing a self-driving and/or assistive driving experience, and progressively transform into interactive agents with some level of autonomy, as well as some context-dependent social features. This introduces new challenges and questions, already relevant in other areas of human-robot interaction (HRI), such as: if an AV is perceived as a social agent by the human with whom it is interacting or collaborating, how are the various facets of its interface and behaviour impacting its human partner? And how do we foster a successful collaboration between the human driver and the AV, maximizing the driver’s comfort, agency, and trust in the AV? Our specific research goal in this area is to explore how the human’s perception of the AV can vary across different levels of information sharing and autonomy of the AV. More precisely, in this work we sought to understand the various factors that could influence naïve participants’ acceptance and trustworthiness of AV. In a between-subjects online study, informed by participatory design, we investigated the effects of different AV interfaces with different information levels on participants’ perceptions of the AV, specifically their trust and comfort ratings. We also sought to understand how much the environment and the AV’s behaviour play a role in determining the level of autonomy users will grant to the AV. We found that the transparency of the AV (described by the amount of information shared with the users) had a significant impact on people’s trust and comfort in the car. Participants’ relationship with the AV’s autonomy however was more complex, and was influenced both by the AV’s transparency, but also by the driving environment and the specific events happening in the scenes.

## 1 Introduction

As their technology progresses, autonomous vehicles (AVs) evolve from augmented technology to increasingly more complex agents, required to collaborate with humans as well as with other artificial agents in multi-agent systems (Ferber and Weiss, 1999; Müller et al., 2016; Schwarting et al., 2019). AVs become more autonomous in their navigation, perception, and decision-making, which necessitates an ability to perceive and understand the other agents’ intentions and states, to communicate their own intentions, and coordinate their actions (Beydoun et al., 2009; Tanevska et al., 2023; Malik et al., 2021; Lee et al., 2021).

The participation of humans in this sort of multi-agent system brings up additional relevant factors into the equation, including safety, transparency, predictability, and trust (Ala-Pietilä et al., 2020; Christ et al., 2016; Okamura and Yamada, 2020). To create an efficient and safe human-AV interaction that the users will perceive as comfortable and trustworthy, it is necessary to understand how the AV’s interface and behavior affects naïve participants’ experience with it (Sripada et al., 2021; Rettenmaier and Bengler, 2021). Moreover, considering that AVs are by design artificial agents created to operate with certain levels of autonomy, it becomes vital to also include their autonomy as a factor when planning how to build a trustworthy and ethical human-AV interaction (Khavas et al., 2020; Gao et al., 2019).

Participatory and user-centered design is a typical approach for the design of interactive agent behaviors which are both functional and socially acceptable (see, e.g., Winkle et al. (2021)). We suggest that this approach is appropriate and important for AVs as they become increasingly autonomous and interactive (Müller et al., 2016; Schwarting et al., 2019). As the AVs would need to interpret and respond to human behavior in real time, effective interaction with human users (drivers, pedestrians, cyclists) becomes essential for ensuring the safety of all participants, and maintaining the users’ trust in the autonomous systems (Detjen et al., 2021; Raats et al., 2020).

In our research, we grounded our study design in the existing EU guidelines on trustworthy and ethical interaction with artificial agents - the ALTAI (Ala-Pietilä et al., 2020), and then proceed to explore specifically the trustworthy dimensions of transparency and autonomy. We wished to understand the interaction between the AV’s designed transparency (corresponding to the amount of information about its processing the AV could share with the users), and the users’ self-reported trust and comfort in the interaction, as well as the amount of autonomy they would confer to the AV.

We began by first conducting a small participatory design study (Tanevska et al., 2023) with potential end users of the AV, in which we discussed two different interaction scenarios, analyzed the information the AV was processing and using in its decision-making, and developed the users’ requirements for a trustworthy interaction. The participatory workshop consisted of two different driver-AV interaction scenarios, where we worked together with the participants to develop different interfaces for the AV for their preferred levels of transparency, as well as analyzed how comfortable they were with the different levels of autonomy (LoA) in which the AV functioned. The purpose of the participatory workshop was to 1) validate the two different interaction scenarios, 2) understand the preference of human users’ with respect to the AV’s autonomy in different scenarios, and 3) design the AV’s interface and the amount of information it can communicate.

We then followed the participatory workshop with a larger-scale online study, whose design was informed by the findings of our workshop. With it, we wished to further investigate how the AV’s transparency and autonomy interact with the users’ sense of trust and comfort, as well as analyze any correlations between participants’ demographics and driving experience, and their perception of the AV.

The rest of this paper is organized as follows: Section 2 gives an overview of the related work in the field of trustworthy human-AV interaction; Section 3 presents our experimental materials and methods; Section 4 discusses the results from our study; and Section 5 concludes the paper, outlining our next steps.

## 2 Related work

In the context of human-AV interaction, one of the most important challenges in building trustworthiness is ensuring effective human-agent communication. Interpreting and responding to human behavior in real time is key for an effective interaction with human users (be they drivers, pedestrians, or cyclists), which also ensures the safety of all participants, and maintains the users’ trust in the autonomous vehicles. When researching what had been done in the field of Trustworthy Human-AV interaction, we came across a few other works that investigated the various factors impacting trustworthiness, but we also noted a lack of research that involved actively the end users during the design and implementation process, as well as research that explored specifically the users’ autonomy preferences.

Drawing a parallel with the issues we tackle in our work, we grouped the related studies according to two main topics - works exploring the relationship between Trust and Information in human-AV interaction, and works dealing with different methodologies and measurements for Trust in AVs. We did not however find a large enough body of works that explored the concept of autonomy in human-AV interaction in terms of the users’ preferences, and the only other study mentioning user preference and the standardized levels of autonomy was the work by Lee and Samuel (2024), who worked on creating machine learning (ML) models to automatically discern user preferences based on survey data they collected.

### 2.1 Relationship between trust and information

There have been a few other studies in recent years which investigated how the different levels of information communicated to the human users of the AV can influence the trust in the interaction. Mackay et al. (2020) employed a driving simulator to evaluate drivers’ trust in an AV depending on the feedback the vehicle provided, which could be feedback regarding the surrounding vehicles, or feedback regarding the surrounding vehicles and the vehicle’s own decisions. Their findings showed that the system was evaluated as trustworthy and safe regardless of the amount and type of feedback provided. In a similar vein, Koo et al. (2015) explored how the content of the verbalized message accompanying the car’s autonomous action can affect the driver’s attitude and safety performance. Using a driving simulator with an auto-braking function, they tested different messages that provided advance explanation of the car’s imminent autonomous action. Messages providing only “how” information describing actions (e.g., “The car is braking”) led to poor driving performance, whereas “why” information describing reasoning for actions (e.g., “Obstacle ahead”) was preferred by drivers and led to better driving performance. Providing both “how and why” resulted in the safest driving performance but increased negative feelings in drivers.

Adding another dimension to the information level and including risk in the equation, Ha et al. (2020) examined the effects of explanation types and perceived risk on trust in autonomous vehicles, using three types of explanations (i.e., no, simple, and attributional explanations), and four autonomous driving situations with different levels of risk. Their results showed that explanation type significantly affected trust in autonomous vehicles, and the perceived risk of driving situations significantly moderated the effect of the explanation type. At a high level of perceived risk, attributional explanations and no explanations led to the lowest and highest values in trust, respectively. However, at a low level of perceived risk, these effects reversed.

Finally, looking at a different user group (cyclists), Hou et al. (2020) conducted research on cyclist-AV interaction, starting with preliminary participatory design studies which informed the implementation of an immersive VR AV-cyclist simulator, and the design and evaluation of a number of AV-cyclist interfaces. Their findings suggested that AV-cyclist interfaces can improve rider confidence in lane merging scenarios, but also emphasized the risks over-reliance can pose to cyclists.

### 2.2 Methodologies and measurements for trust in AVs

When we looked at the state of art in trustworthy human-AV interaction, we found no one consistent theoretical model about measuring the various dimensions of trust. Relying on some of the most common measures, Sheng et al. (2019) designed and conducted study where participants were asked to enter their trust level in a Likert scale in real-time during experiments on a driving simulator. They also collected physiological data (e.g., heart rate, pupil size) of participants as complementary indicators of trust. Their results showed that the missing automation alarms had a significant impact on humans’ trust while driving mode whereas weather conditions did not.

Similarly using physiological information, Morra et al. (2019) proposed a methodology to validate the user experience in AVs based on continuous, objective information gathered from physiological signals, while the user was immersed in a Virtual Reality-based driving simulation. They obtained qualitative and quantitative evidence that a complete picture of the vehicle’s surrounding, despite the higher cognitive load, is conducive to a less stressful experience. Additionally, after having been exposed to a more informative interface, users involved in the study were also more willing to test a real AV.

Exploring a more advanced factor, Petersen et al. (2019) argued for enhancing situational awareness, which could lead to an increase in the driver’s trust in automation. They manipulated drivers’ situational awareness by providing them with different types of information, such as a status update, or a status update and a suggested course of action. Their findings showed that situational awareness both promoted and moderated the impact of trust in the automated vehicle, leading to better secondary task performance. This result was evident in measures of self-reported trust and trusting behavior. Shahrdar et al. (2019) worked on a new approach for real-time trust measurement between passengers and self-driving cars (SDCs). Using a new structured data collection approach along with a virtual reality SDC simulator, they aimed to understand how various autonomous driving scenarios can increase or decrease human trust and how trust can be re-built in the case of incidental failures. The results of an empirical experiment indicated that most subjects could rebuild trust during a reasonable time frame after the system demonstrated faulty behavior. The autonomous driving style directly influenced the trust of the passengers in the system, with aggressive driving diminishing trust, and defensive/predictable driving increasing trust.

### 2.3 Summary

While there have been several works in the past several years recognizing the importance of Trust in Human-AV Interaction, more often than not this research has not adequately involved the potential users themselves in the design and implementation of the interaction scenarios and/or interfaces. At the time of our study’s design, the only other work we could find which dealt with both Trust in AV and Participatory Design was the research conducted by Hou et al. (2020), which however had the application of cyclist-AV interaction.

Similarly, we did not find a body of related works that investigated explicitly autonomy preferences, except for the work by Lee and Samuel (2024) which utilized the standardized levels of autonomy for AVs to explore users’ preferences and design a predictive ML model.

With our contribution, we aim to add to the body of research exploring transparency and autonomy, as well as bridge the gap between user-centered design and trustworthy human-AV interaction, and advocate for iterative studies as well as for more proactive involvement of the final users in the design process.

## 3 Materials and methods

The two main dimensions of trustworthiness we wished to explore in our research were transparency and autonomy, as well as their overall relation with users’ trust and comfort. We mapped the transparency dimension to the amount of information the AV could share with the human users about its processing and decision-making; while the autonomy dimension was intuitively represented by the level of autonomy (LoA) the users would confer to the car.

To address how these concepts interact with each other, we formulated the following research questions.

- RQ 1 What is the effect of the designed transparency levels (High Information vs. Low Information) on the interaction with the AV?
  - RQ 1.1 Does a higher level of transparency lead to higher user ratings of trust and comfort?
  - RQ 1.2 Does a higher level of transparency lead to higher levels of autonomy conferred to the AV?
- RQ 2 What is the effect of the external factors (such as the driving environment and potential high-risk scenes) on the interaction with the AV?
  - RQ 2.1 Is there a significant difference in the user ratings of trust and comfort across different scenarios and scenes?
  - RQ 2.2 Is there a significant difference in the levels of autonomy conferred to the AV across different scenarios and scenes?

### 3.1 Study background and design

Prior to designing the large-scale online study, we developed the interaction scenario and validated the metrics during an in-person co-designing participatory workshop (Tanevska et al., 2023). We created two different driver-AV interaction scenarios, and worked together with the workshop participants to develop different interfaces for the AV for their preferred levels of information sharing (which then evolved into our study’s transparency levels), as well as to understand how comfortable they would be with the different levels of autonomy (LoA) in which the AV functioned (which was retained as a metric also in the online study).

**FIGURE 1:**
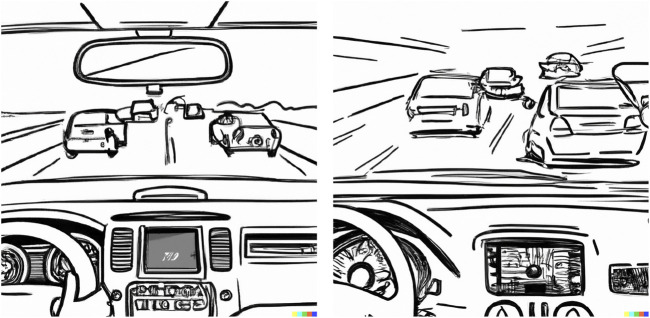
Highway driver-AV scenario. AI-generated images of a car’s dashboard with the driver’s POV, car driving on a busy highway and another car cutting in from the right.

**FIGURE 2:**
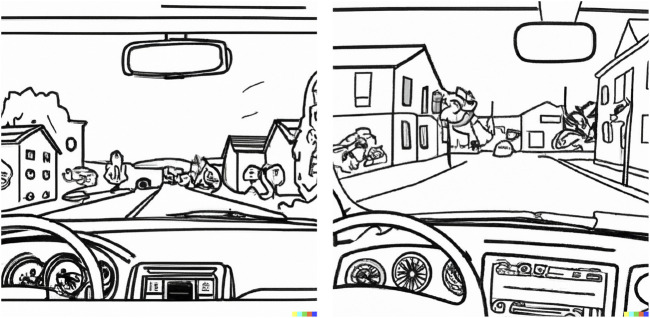
Suburbs driver-AV scenario. AI-generated images of a car’s dashboard with the driver’s POV, car driving on an empty neighborhood street, with a school building coming up on the left.

The two use case scenarios were presented to participants as series of computer-generated images^1^ on a flip-chart, and participants were tasked to imagine themselves in the role of the driver of the AV. Both scenarios were designed with three scenes, where the second scene presented an unpredictable (and/or potentially high-risk) moment. In the first scenario (Figure 1), the AV was driving along the middle lane on a busy highway, when another car unpredictably merged from the right at an unsafe distance and speed, creating an imminent collision risk. As the scenario progressed, the AV employed the emergency braking procedure. In the second scenario (Figure 2), the AV was driving down an unfamiliar empty suburban street, when it suddenly slowed down to 20 km/h without any visible obstacle. As the scenario progressed, a school that was previously obscured behind trees came into view.

The levels of autonomy we worked with in this study were the SAE Levels of Driving Automation (SAE International Recommended Practice, 2018), specifically Levels 0 through 3, i.e.,.

- 0. Driver (the person is in full control of the AV)
- 1. Feet off (the person has to keep their hands on the wheel and steer, but there’s some automated features)
- 2. Hands off (the person may remove their hands from the wheel, but need to keep their eyes on the road at all times)
- 3. Eyes off (the person may also do other activities in the AV, as long as they’re mentally present and ready to take over if needed)

These levels were selected as they were at the time the ones most commonly present in the commercially-available AVs on the market. For each of the three scenes in both scenarios, participants were asked to select the highest level of autonomy they were comfortable with.

At the same time, participants were presented with all of the information the AV uses in its calculations of the risk in the scene and its next actions. This included information such as the state of the AV (its speed, GPS location, etc.), the state of the environment (the static and dynamic objects in it and how they were interacting with the AV), and the traffic rules currently active in the scene.

Participants were then asked to work together as a group to design an interface for the AV (using post-it notes and drawing on the scenario images on the flip-chart), and decide on the amount and type of information shown. The resulting interface was then considered throughout the scenes in both scenario, and any eventual modifications were made during the scene changes.

Two distinct interface designs were the outcome of this activity: 1) a minimal interface similar to modern car GPS navigators, without any additional information on the AV’s decision-making processes; and 2) a much more detailed and complex design, containing visual depiction of all of the information the AV is processing and its upcoming actions. The participants who were in favor of the first design tended to want explicit communication from the AV only in an emergency, whereas the ones that favored the second design wished to be able at any moment to see how the AV is processing and understanding the scene, and how it selects its actions, seeing it as crucial for the development of their trust in it.

In a previous workshop paper we discussed in-depth the details of the participatory design study and its findings (Tanevska et al., 2023). The biggest relevance to this work were the two distinct interfaces, which informed the design of our large-scale online study (presented in the rest of this section) and became the two information levels - with the minimal interface giving us the Low Information condition, and the complete interface the High Information one.

### 3.2 Participants

In total 206 participants took part in our study, equally distributed between male and female^2^, and their age ranged from 20 to 73 years (M = 36.42, SD = 13.185).

Participants were recruited via the crowd-sourcing platform Prolific^3^. We used the platform’s filters to recruit English-speaking participants with a valid driver’s license. During the recruitment process we initially collected responses from 249 potential participants, however out of these 43 were then excluded from the study for failing the attention or validity checks (more details in Section 3.4).

From the recruited participants, 29.1% had been active drivers for over 20 years, 18.9% between 11 and 20 years, 42.2% between 3 and 10 years, and 9.7% had been driving less than 2 years. Regarding the frequency of driving, 66% of participants drove daily, 28.6% drove several times a week, and the rest were occasional drivers, with only 2 participants no longer being active drivers.

The study received ethical approval by the Swedish Ethical Review Authority (Etikprövningsmyndigheten, application code 2023–01688-01); specifically the application was evaluated as “not needing an ethical approval” due to the research not falling under the provisions specified in Section 4 of the Ethical Review Act of Sweden. No underaged or vulnerable participants were involved; informed consent forms were present and complete.

### 3.3 Experimental design and protocol

Our study followed a 2 × 2 between-subjects design, with the two levels corresponding to the designed information level provided by the AV’s interface (High Information vs Low Information) and the order in which participants experienced the two scenarios (Highway-first vs Suburbs-first). Participants were randomly assigned to one of the four groups.

At the start of the study, participants completed questionnaires on demographics, as well as an adapted, abridged version of the Driver Behaviour questionnaire (DBQ) (Reason et al., 1990). A brief slide presentation on AVs followed, which introduced participants to the term, outlined the types of information the AV processes when making its decisions, and presented participants with the concepts on autonomy in AVs and how it’s quantified. It also broached the concept of social interaction with AVs, and what it entails.

After the presentation, there was an attention check (describing one SAE autonomy level and asking participants to select the correct one), after which participants filled the last questionnaire, an AV-centered adaptation of the NARS questionnaire (Nomura et al., 2004), aimed at identifying and quantifying any negative attitudes or preconceptions participants may have towards AVs. Our version (referred to as AV-NARS) had 10 items, adapted to fit the context of interaction with AVs, e.g., I would feel nervous driving an autonomous car while other people could observe me. Instead of the original I would feel nervous operating a robot in front of other people.

After the questionnaires, all participants were presented with the same two scenarios (Highway and Suburbs) from the participatory design workshop as described in Sect. 3.1, but with their order varied depending on which experimental group they were assigned to. Both scenarios consisted of three scenes, with the second scene being the one with the unexpected moment (the second car merging unsafely, or the AV inexplicably slowing down). Half of the participants experienced the two scenarios with a Low Information interface, and the other half with a High Information one.

**FIGURE 3:**
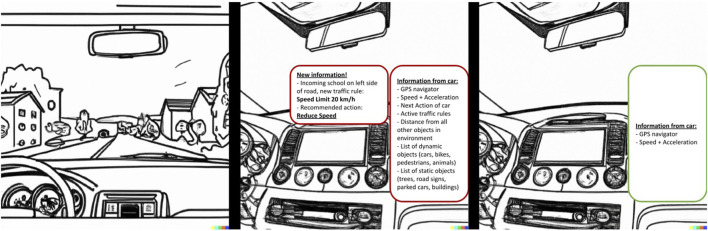
Suburbs driver-AV scenario in Scene 2, comparing the High Information and Low Information interfaces. The left panel shows an AI-generated image of a car’s dashboard with the driver’s POV, the car driving on an empty neighborhood street, with a school building coming up on the left. The middle and right panels show the High Information (HI) and Low Information (LI) interfaces respectively, with the HI interface presenting the full list of data processed by the AV, plus the recommended action, and the LI interface presenting only the GPS navigator and information on the speed.

Each of the three scenes consisted of the same computer-generated image of the dashboard view of the scene as mentioned in Section 3.1, but this time merged with an overview of the information presented to them (see Figure 3 for an example illustration and comparison between the two interfaces), and with a roleplay-like narration of what happens in the scene added below.

Participants were then asked “What do you do?” and could select from multiple-choice answers, where each of their action choices corresponded to a Level of Autonomy between 0 and 3. After they selected an action, participants answered questions on their comfort levels with the situation, and their trust in the AV. The questions are described in greater detail in the following section.

### 3.4 Data collection

Two platforms were used in the data collection process in our study, and the data collection took place in late 2023 and early 2024. The majority of the participant demographics data was collected via the recruiting platform Prolific^4^, and the remaining questionnaires and user behaviour data was collected via Uppsala University’s internal GDPR-compliant survey platform Kurt^5^.

During the data collection process, we included an attention check in the Kurt survey, and a validity check on Prolific for the amount of time participants took to complete the study. The 43 submissions which either failed the attention check, or took a significantly longer or shorter time to complete (e.g., 3 h or 3 min, as opposed to the average 20–30 min) were excluded from the study.

#### 3.4.1 Demographic data

Through Prolific’s recruitment process, we obtained general demographic data from our participants, including their age, legal sex, country of residence, and employment status. Additionally, at the start of the survey in our own platform, participants had the option to declare their gender identity.

We then collected information on participants’ driving experience, specifically how long they have had their driving license, how long they have been an active driver, their driving frequency and their usual driving environment. The full list of driving items is shown in Table 1.

**TABLE 1:** Driving experience items.

Item	Question	Answer(s)
Driving license	For how many years have you had a driving licence?	Less than 1 year
		1–2 years
		3–5 years
		6–10 years
		11–20 years
		More than 20 years
Active driving	From those, how many years have you been an active driver?	Less than 1 year
		1–2 years
		3–5 years
		6–10 years
		11–20 years
		More than 20 years
Driving frequency	How frequently do you drive?	Daily
		A few days a week
		A few days a month
		A few days a year
		I no longer drive
		Other (please describe)
Driving environment	Select your usual driving environments: (multiple options can be selected)	Highway
		City center/urban area
		Suburban neighborhood area
		City outskirts/rural area
		Other

#### 3.4.2 Questionnaires

In addition to the demographic and driving experience items, participants filled two more questionnaires, as mentioned in Section 3.3. These were an adapted and abridged version of the Driver Behaviour questionnaire (DBQ) (Reason et al., 1990) and an AV-centered adaptation of the NARS questionnaire (Nomura et al., 2004), aimed at measuring negative attitudes towards AVs. The full list of items for both of these questionnaires is shown in Table 2.

**TABLE 2:** DBQ and AV-NARS questionnaire items, asked once at the start of the experiment.

Metric	Item
DBQ	1. Check your speedometer and discover that you are unknowingly traveling faster than the legal limit
	2. Distracted or preoccupied, realize belatedly that the vehicle ahead has stopped, and have to slam on the brakes to avoid a collision
	3. Merging onto a main road, you turn into the path of an oncoming vehicle that you hadn’t seen, or whose speed you had misjudged
	4. Stuck behind a slow-moving vehicle on a two-lane highway, you are driven by frustration to try and overtake it in risky circumstances
	5. Try to overtake without first checking your mirror, and then get honked at by the car behind which has already begun its overtaking maneuver
	6. Deliberately disregard the speed limits late at night or very early in the morning
	7. Lost in thought or distracted, you fail to notice a person waiting at a pedestrian crossing who has right of way
	8. Ignore “give way” rules, and narrowly avoid colliding with traffic that has right of way
	9. Drive with only “half an eye” on the road while looking at your phone, a map, changing the radio station*etc.*
	10. Get in unofficial “races” with other drivers
AV-NARS	1. I would feel uneasy if autonomous cars could recognize my emotions
	2. Something bad might happen if autonomous cars are programmed with social skills
	3. I would feel relaxed if my autonomous car talked with me
	4. I would feel uneasy if I was given a job where I had to drive autonomous cars
	5. I would feel nervous driving an autonomous car while other people could observe me
	6. I would hate the idea that autonomous cars are capable of making judgments about things
	7. I would feel very nervous standing in front of an autonomous car
	8. I feel that if I depend on autonomous cars too much, something bad might happen
	9. I would feel paranoid talking with my autonomous car
	10. I think that in the future autonomous cars will be a lot more prevalent in society and will replace all other cars, and I am afraid of that possibility

The adapted DBQ consisted of ten examples of mistakes and violations that people have experienced while driving. For each item, participants were asked to indicate how often (if at all) they had engaged in that behavior over the last 5 years (or less, if they had been driving for less time). The ratings were on a 0–5 scale, with the following meanings: [0 = Never; 1 = Hardly ever; 2 = Occasionally; 3 = Quite often; 4 = Frequently; 5 = Nearly all the time; X = Not applicable]. The adapted DBQ items showed acceptable internal consistency in this study, with Cronbach’s alpha = 0.772.

The second questionnaire was the adapted NARS (referred to later as AV-NARS), transformed for identifying and quantifying any negative attitudes or preconceptions participants may have towards AVs. Participants were presented with ten statements, meant to describe their attitude towards autonomous cars, and asked to rate them on a scale from 1 to 5, where the numbers corresponded to the following meanings [1 = I strongly disagree; 2 = I disagree; 3 = Neutral; 4 = I agree; 5 = I strongly agree]. This questionnaire was first piloted and validated in our earlier participatory workshop (Tanevska et al., 2023), where despite having only 11 participants, the scale got a Cronbach’s alpha value of 0.71, giving it an acceptable internal consistency. In this study instead, the Cronbach’s alpha increased to 0.854, giving the scale a good internal consistency.

#### 3.4.3 Action choice (level of autonomy)

The selection of action following each of the six scenes gave us the Level of Autonomy (LoA) ratings. These questions were a multiple-choice questions where each choice corresponded to a LoA between 0 and 3 (see Table 3). This was averaged in the cases where participants chose more than one option, with the final LoA score thus being a decimal value ranging from 0 to 3.

**TABLE 3:** Repeated questionnaire items asked after each interaction scene.

Metric	Question	Answer(s)
Action (LoA)	What do you do? (multiple options can be selected)	I do nothing
		I let the car continue navigating
		I quickly check my phone for messages
		I check out the area around me and look around for anything interesting
		I focus my eyes on the road and check for any people or animals that may approach the road
		I put my hands back on the wheel
		I take over the driving control from the car
Comfort	How do you feel about the situation?	Relaxed, calm
		Anxious, tense
		Neutral
		Unsure
Trust	Please select the statements you agree with: (multiple options can be selected)	I feel that the car is reliable
		I can trust the car’s actions to be safe
		I don’t feel safe in the car
		I can rely on the car to make safe decisions for my wellbeing
		I feel I can follow the car’s instructions
		I don’t trust the behaviour of the car
		I don’t agree with any of these (please explain)

To provide a concrete example for Scene 1, possible answers were: I do nothing (Level 3), I let the car continue navigating (Level 3), I quickly check my phone for messages (Level 3), I check out the area around me and look around for anything interesting (Level 2), I focus my eyes on the road and check for any people or animals that may approach the road (Level 2), I put my hands back on the wheel (Level 1), I take over the driving control from the car (Level 0).

#### 3.4.4 Comfort and trust in the AV

As we did not find a standardized measurement of trust in the specific context of human-AV interaction (as discussed also in Section 2), we looked at several well-known scales used in the field of human-robot interaction, and extracted the relevant items for use in our scenario.

We started from the works of Bartneck et al. (2009) and Hancock et al. (2021), and explored the Godspeed’s measurements of perceived safety, as well as the updated set of robot factors of performance and dependability. We also looked at a few other scales on competency and trust in human-robot interaction, such as the items from the Human-Robot Trust Scale (Pinto et al., 2022), the research by Calvo-Barajas et al. (2024), and the design guidelines for trustworthy human-agent interaction by Barajas et al. (2025). We ultimately defined one measurement of the user comfort, and a combined measurement for the user trust, consisting of a few metrics on the perceived competence and safety.

For expressing their comfort level with the current situation, we implemented the states used in the Godspeed item on perceived safety (Bartneck et al., 2009). Participants were asked a single-choice question on how they felt about the situation, where they could answer relaxed, calm (rated 1), anxious, tense (rated −1), neutral, or unsure (both rated 0).

For their level of trust in the car instead, participants were asked to describe how they felt via a multiple choice question. The answers were coded as 1 or -1 depending on whether they expressed a positive or negative trust sentiment, and then summed up to form the total Trust score, which ranged from −2 to 4. Participants could also provide a free-text answer, if they felt that none of the statements were accurate about the current situation and their decision^6^.

The items used in the Trust question were selected and adapted from the scales mentioned above as the most relevant for the scenario of interaction with AV (i.e., items that included social interaction, reciprocity, theory of mind, etc., were excluded as they were deemed non-applicable to the scenario in question). The complete details of the Comfort and Trust metrics are shown in full in Table 3.

## 4 Results

In this study, we were interested in exploring trustworthiness in the context of driver-AV interaction, and more specifically its two dimensions of transparency and autonomy and their overall relation with users’ trust and comfort.

We asked the following research questions.

- RQ 1 What is the effect of the designed transparency levels (High Information vs. Low Information) on the interaction with the AV?
  - RQ 1.1 Does a higher level of transparency lead to higher user ratings of trust and comfort?
  - RQ 1.2 Does a higher level of transparency lead to higher levels of autonomy conferred to the AV?
- RQ 2 What is the effect of the external factors (such as the driving environment and potential high-risk scenes) on the interaction with the AV?
  - RQ 2.1 Is there a significant difference in the user ratings of trust and comfort across different scenarios and scenes?
  - RQ 2.2 Is there a significant difference in the levels of autonomy conferred to the AV across different scenarios and scenes?

For each of these four RQs we performed a series of confirmatory statistical tests, presented in Sections 4.1–4.4. Additionally, we also performed a statistical exploratory analysis of participants’ demographic data and driving experience, to understand if there were any correlations between the demographic data, the results of the DBQ and AV-NARS questionnaires, and the participant ratings of the Levels of Autonomy (LoA) and Trust in the AV. This exploratory analysis is presented in Section 4.5. All analysis was conducted using the Jamovi statistical software^7^.

A Shapiro–Wilk test for normality showed that our data deviated from a normal distribution for all dependent variables. We then used non-parametric statistical analysis, specifically the Mann–Whitney U-test, the Wilcoxon’s signed rank test, and Friedman’s ANOVA, to analyze our data. The post-hoc analysis in the Friedman ANOVA tests was performed with the Pairwise signed-ranks tests using the Durbin test (named the Durbin-Conover method in Jamovi). Effect size was reported using the rank-biserial correlation coefficient for the Mann-Whitney U test and the Wilcoxon’s signed rank test, and Kendall’s W for Friedman’s ANOVA.

We will now go over every RQ individually in the following subsections and present our findings. We also summarize our key findings in Table 4, mapping the four research questions to the outcomes.

**TABLE 4:** Summary of the key findings, divided by research questions.

Research question	Result	Key takeaway	Effect size
RQ 1.1: Does a higher level of transparency lead to higher user ratings of trust and comfort?	Hypothesis confirmed	The designed Transparency affects to some extent how participants’ Trust and Comfort evolve during the scenario	Small-to-moderate
RQ 1.2: Does a higher level of transparency lead to higher levels of autonomy conferred to the AV?	Hypothesis rejected	The designed Transparency has almost no effect on the Level of Autonomy	Negligible
RQ 2.1: Is there a significant difference in the user ratings of trust and comfort across different scenarios and scenes?	Hypothesis confirmed	The scenario and driving environment affect clearly the user ratings of Trust and Comfort	Moderate-to-large
RQ 2.2: Is there a significant difference in the levels of autonomy conferred to the AV across different scenarios and scenes?	Hypothesis confirmed	The scenario and driving environment have a clear impact on the conferred Level of Autonomy	Large

### 4.1 Transparency and user trust and comfort

With RQ 1.1 we hypothesized that a higher Transparency level would lead to higher reported Trust and Comfort by the users. We conducted independent sample Mann–Whitney U-tests to compare the differences in ratings between the High Information (HI) and Low Information (LI) participants for the Comfort and Trust ratings, and report the effect sizes using the rank-biserial correlation coefficient $r_{rb}$ .

**FIGURE 4:**
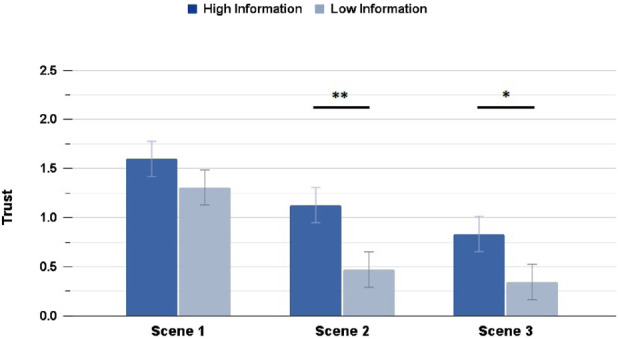
Trust ratings in the Highway scenario: interacting with a High Information interface resulted in significantly higher rating in Scenes 2 and 3 (∗ = *p* < 0.05, ∗∗ = *p* < 0.01, ∗∗∗ = *p* < 0.001).

**FIGURE 5:**
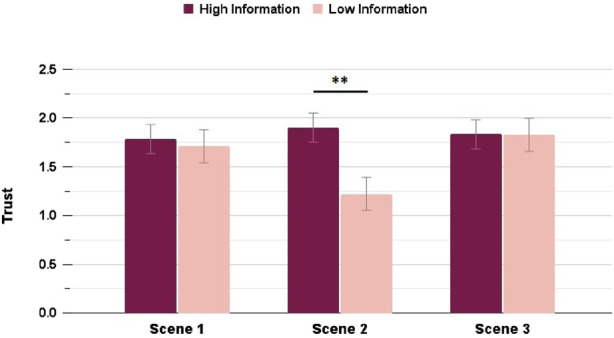
Trust ratings in the Suburbs scenario: interacting with a High Information interface resulted in significantly higher rating in Scene 2 only (∗ = *p* < 0.05, ∗∗ = *p* < 0.01, ∗∗∗ = *p* < 0.001).

Trust ratings showed significant differences (with a small effect size) between HI and LI in the Highway scenario, both across the average Trust rating $\left(p=0.0017,r_{rb}=0.171\right)$ , and for the Trust ratings in Scene 2 $\left(p=0.006,r_{rb}=0.201\right)$ and Scene 3 $\left(p=0.023,r_{rb}=0.159\right)$ as well. The Suburbs condition instead had no significant differences in the average Trust rating (p=0.117) , and only had a significantly different rating with a small-medium effect size in Scene 2, when the unexpected moment happens $\left(p=0.003,r_{rb}=0.214\right)$ . Figures 4, 5 shows the differences in the HI and LI ratings for all Highway and Suburbs scenes, respectively.

**FIGURE 6:**
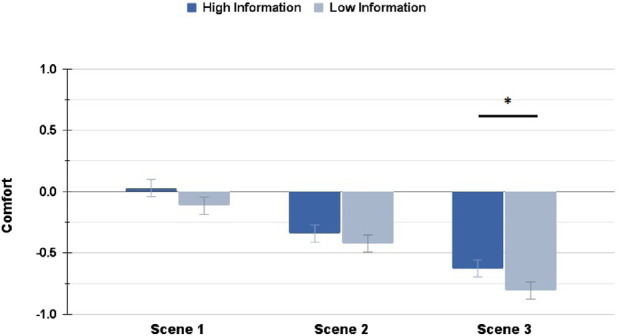
Comfort ratings in the Highway scenario: interacting with a High Information interface resulted in significantly higher rating in Scene 3 only (∗ = *p* < 0.05, ∗∗ = *p* < 0.01, ∗∗∗ = *p* < 0.001).

**FIGURE 7:**
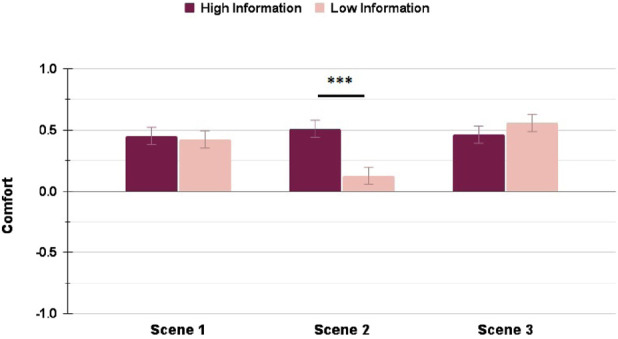
Comfort ratings in the Suburbs scenario: interacting with a High Information interface resulted in significantly higher rating in Scene 2 only (∗ = *p* < 0.05, ∗∗ = *p* < 0.01, ∗∗∗ = *p* < 0.001).

Similarly, the Comfort ratings showed significant differences (with a small effect size) between HI and LI conditions in the Highway scenario for the average Comfort rating $\left(p=0.048,r_{rb}=0.132\right)$ , and for the Comfort rating in Scene 3 $\left(p=0.027,r_{rb}=0.109\right)$ . In the Suburbs condition, the Transparency levels had a small effect on the average Comfort rating $\left(p=0.048,r_{rb}=0.131\right)$ and a moderate effect on the Comfort rating in Scene 2 $\left(p<0.001,r_{rb}=0.278\right)$ . Figures 6, 7 shows the differences in the HI and LI ratings for all Highway and Suburbs scenes, respectively.

Overall, the Transparency level had an effect on the user Trust and Comfort, but with different trends for the different scenarios. In the Highway scenario, the user ratings of Trust and Comfort dropped almost linearly through the three scenes, but with the LI participants experiencing a starker drop through Scenes 2 and 3 - the high-risk moment and the resolution. On the other hand, in the Suburbs scenario the values seemed to be much more stable, with only the LI participants experiencing a drop during Scene 2, but then recovering in Scene 3, whereas the HI participants’ ratings remained stable across all scenes.

These findings to a large extent confirmed RQ 1.1 - while we could find no differences in the starting values of Trust and Comfort in the first scene between HI and LI, the information level did play a role in the remaining part of the scenario, and how participants’ Trust and Comfort changed.

### 4.2 Transparency and conferred LoA

**FIGURE 8:**
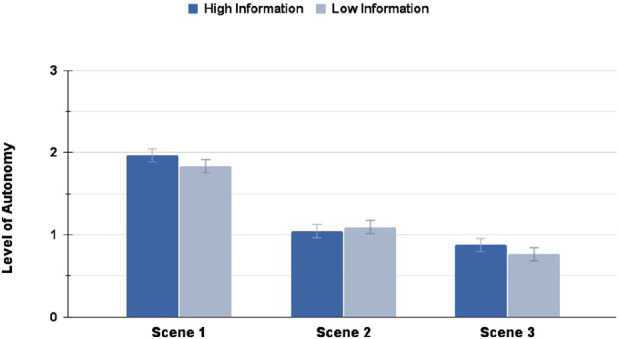
LoA ratings in the Highway scenario: interacting with a High Information interface did not result in any significantly different LoA ratings across the scenes.

With RQ 1.2 we hypothesized that a higher Transparency level would also lead to a higher conferred Level of Autonomy (LoA) by the users to the AV. The Mann–Whitney U-tests found no significant differences between the HI and LI conditions for any of the scenes in the Highway scenario, as seen in Figure 8.

**FIGURE 9:**
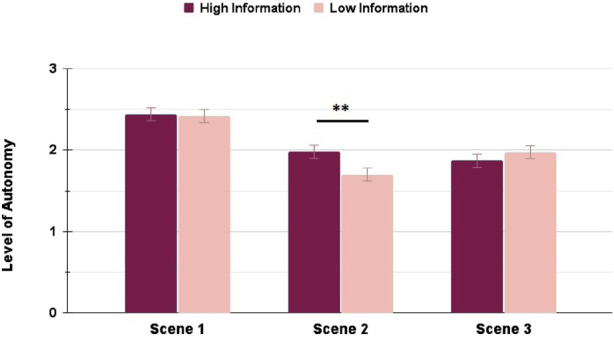
LoA ratings in the Suburbs scenario: interacting with a High Information interface resulted in significantly higher LoA rating in Scene 2 only (∗ = *p* < 0.05, ∗∗ = *p* < 0.01, ∗∗∗ = *p* < 0.001).

In the Suburbs scenario instead, the only significant difference with a small effect size in the LoA rating could be seen in Scene 2 $\left(p=0.004,r_{rb}=0.210\right)$ , as shown in Figure 9, with the LI condition experiencing a bigger drop in the LoA rating, similarly to the findings in 4.1.

These findings mostly reject the hypothesis from RQ 1.2. The Level of Autonomy seems to be a metric which is highly dependent on the interaction scenario and environment, and almost not at all on the designed Transparency. We thus proceeded to explore the effect of the environment on the user behaviour and reported metrics.

### 4.3 The environment and user trust and comfort

With the two hypotheses in RQ 2, we wished to understand how the different environments of interaction (Highway vs Suburbs) impacted the trustworthy metrics, as well as explore the trends across the individual scenario scenes.

To investigate RQ 2.1 and RQ 2.2, we conducted three types of analysis. We first performed a series of independent sample Mann–Whitney U-tests across the chronological data, comparing the values in Scenario 1 and 2 for the two different Order levels - the participants which experienced first the Highway and then the Suburbs scenario (referred to as the HS group), and the participants which had the inverse order of Scenarios (the SH group). We then performed a series of paired sample Wilcoxon’s tests, to investigate the differences in participants’ own Trust and Comfort ratings in the two different scenarios. Finally, we also conducted a Repeated Measures Friedman’s ANOVA to see the difference in the Highway and Suburbs values over the course of the entire scenario (Scene 1–2–3).

#### 4.3.1 Independent comparison between HS and SH groups

The Mann–Whitney U-tests showed significant differences (p<0.001) between the Highway and Suburbs conditions for all Comfort values throughout Scenarios 1 and 2, with an overall large effect size ( $r_{rb}=0.611$ for Scenario 1 and $r_{rb}=0.749$ for Scenario 2). The HS group had significantly lower Comfort ratings in Scenario 1 compared to the SH group, and significantly higher Comfort ratings in Scenario 2.

The Trust ratings had slightly different trends. In Scenario 1, the Mann–Whitney U-tests showed significant differences with a moderate effect (p<0.001) in all Trust ratings except for the Scene 1 rating (p=0.192) , which was the only rating in the first scenario with similar values for all participants (with the mean around 1.3 for the HS group and 1.5 for the SH group). The largest effect size was noted in the Scene 3 comparison $\left(r_{rb}=0.400\right)$ .

This trend was similar for Scenario 2 as well, where in Scene 1 there were again no significant differences in the Trust ratings between the HS and SH groups (p=0.162) , which then changed with the HS ratings dropping significantly, both for Scene 2 $\left(p=0.028,r_{rb}=0.151\right)$ and Scene 3 $\left(p<0.001,r_{rb}=0.341\right)$ , as well as for the average Trust $\left(p=0.002,r_{rb}=0.237\right)$ .

#### 4.3.2 Paired comparison for all participants between the two scenarios

We then investigated how participants’ own ratings of Trust and Comfort compared between their two scenarios.

In the HS group, all participants experienced a significant increase (p<0.001) in their Trust and Comfort ratings between the first and second Scenario, with a large effect size and $r_{rb}$ ranging between −0.952 and −1 for the Comfort ratings, and −0.436 and −0.874 for the Trust ones. The compared pairs were Highway Scene 1 with Suburbs Scene 1, Highway Scene 2 and Suburbs Scene 2, Highway Scene 3 and Suburbs Scene 3, and Highway Average with Suburbs Average.

In the SH group, we could find no significant differences for the first Scene comparison (Suburbs Scene 1 with Highway Scene 1), neither in the Trust (p=0.470) nor in the Comfort (p=0.356) ratings. The remaining paired comparisons (Suburbs Scene 2 with Highway Scene 2, Suburbs Scene 3 with Highway Scene 3, and Suburbs Average with Highway Average) however all showed significant decreases with a large effect size in the Trust $\left(r_{rb}=0.541\right)$ and Comfort $\left(r_{rb}=0.840\right)$ ratings in Highway, with p<0.001 for all comparisons except Scene 2 Trust which was p=0.017 .

#### 4.3.3 Change of values between the scenes in Highway and Suburbs

We conducted a Repeated Measures Friedman’s ANOVA, checking the changes in the Trust and Comfort values separately for the Highway and Suburbs ratings (averaged for the High Information and Low Information groups).

The Friedman’s ANOVA showed a significant difference with a small effect across the separate scenes in the Highway scenario, both for the Trust ( $\chi_{\left(206,2\right)}^{2}=44.5,p<0.001,W=0.108$ ) and Comfort ( $\chi_{\left(206,2\right)}^{2}=114,p<0.001,W=0.277$ ) ratings. The pairwise post-hoc check with the Durbin-Connover method confirmed significant differences (p<0.001) between all three pairs (Scene 1–2, Scene 2–3, and Scene 1–3) for the Comfort ratings, and between the Scene 1-2 and Scene 1-3 for the Trust rating.

In the Suburbs scenario there were also significant differences from the Friedman’s ANOVA with a small effect size, both for the Trust ( $\chi_{\left(206,2\right)}^{2}=7.63,\;p=0.022,W=0.018$ ) and Comfort ( $\chi_{\left(206,2\right)}^{2}=17.1,p<0.001,\;w=0.042$ ) ratings. Specifically, the pairwise post-hoc check with the Durbin-Connover method confirmed significant differences between Scene 2–3 (p=0.006) in the Trust rating, and between Scene 1–2 (p=0.003) and Scene 2–3 (p<0.001) for the Comfort rating.

**FIGURE 10:**
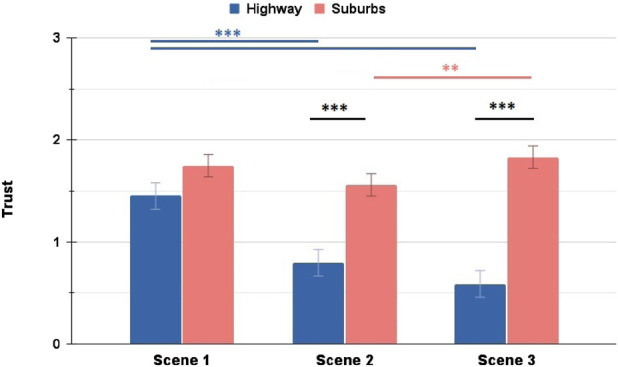
Comparison of Trust ratings between Highway and Suburbs: the Suburbs scenario marked significantly higher ratings for Scenes 2 and 3. Additionally, both scenarios noted significant changes in their ratings between different scenes (∗ = *p* < 0.05, ∗∗ = *p* < 0.01, ∗∗∗ = *p* < 0.001).

**FIGURE 11:**
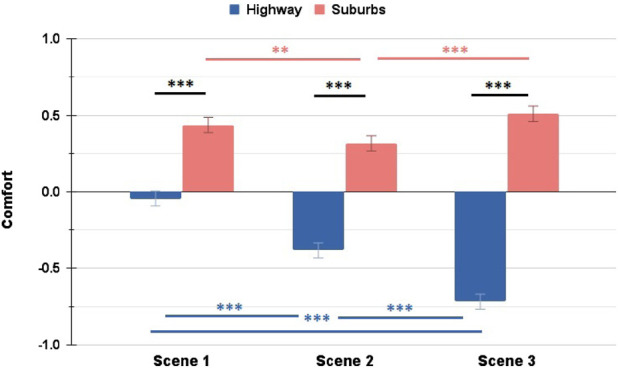
Comparison of Comfort ratings between Highway and Suburbs: the Suburbs scenario marked significantly higher ratings for all three scenes. Additionally, both scenarios noted significant changes in their ratings between different scenes (∗ = *p* < 0.05, ∗∗ = *p* < 0.01, ∗∗∗ = *p* < 0.001).

Overall, the two scenarios show different patterns across the scenes - participants expressed consistently lower ratings of Trust and Comfort during the Highway scenario, whereas during the Suburbs their ratings dropped in the unexpected Scene 2, but then recovered in Scene 3. Figures 10, 11 show these trends. This confirms our hypothesis in RQ 2.1, since the scenario and driving environment affect clearly the user ratings of Trust and Comfort.

### 4.4 The environment and conferred LoA

Finally, for investigating the effect of the environment on the LoA as defined in RQ 2.2, we conducted the same three types of analysis as in Section 4.3.

#### 4.4.1 Independent comparison between HS and SH groups

The Mann–Whitney U-tests had significant differences (p<0.001) with a large effect for all LoA comparisons between Scenario 1 and 2 for the HS and SH groups. Namely, the HS group had a significantly lower conferred LoA in Scenario 1 compared to the SH group (and $r_{rb}$ ranging between 0.356 and 0.619), and significantly higher LoA for Scenario 2 (and $r_{rb}$ ranging between −0.424 and −0.644).

#### 4.4.2 Paired comparison for all participants between the two scenarios

We then investigated how participants’ conferred LoA evolved between their two scenarios using paired sample Wilcoxon’s tests. In the HS group, participants consistently conferred a significantly lower Level of Autonomy ( p<0.001 , and $r_{rb}$ showing a very large effect between −0.787 and −0.945) to the AV in all scenes from the first scenario compared to the second. As in Section 4.3, the compared pairs were Highway Scene 1 with Suburbs Scene 1, Highway Scene 2 and Suburbs Scene 2, Highway Scene 3 and Suburbs Scene 3, and Highway Average with Suburbs Average. The same large effect was also found in the SH group, with all participants having higher values in Suburbs compared to Highway ( p<0.001 , and $r_{rb}$ ranging between 0.532 and 0.864).

#### 4.4.3 Change of values between the scenes in Highway and Suburbs

Finally, we used a Repeated Measures Friedman’s ANOVA to check for the changes throughout the scenes of the LoA ratings, separately for the Highway and Suburbs scenario.

The Friedman’s ANOVA showed a significant difference in the conferred LoA across the separate scenes in the Highway scenario ( $\chi_{\left(206,2\right)}^{2}=197,p<0.001,W=0.478$ ). The pairwise post-hoc check with the Durbin-Connover method confirmed significant differences (p<0.001) between all three pairs (Scene 1–2, Scene 2–3, and Scene 1–3). The Suburbs scenario also showed significant differences in the LoA values ( $\chi_{\left(206,2\right)}^{2}=117,p<0.001,W=0.284$ ), confirmed with the Durbin-Connover post-hoc, with p<0.001 for Scene 1–2 and 1–3, and p=0.012 for Scene 2–3.

**FIGURE 12:**
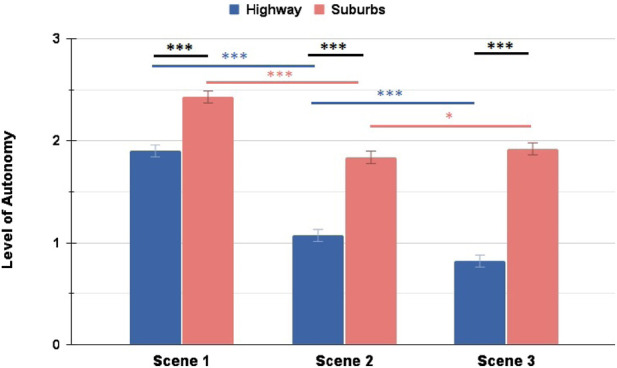
Comparison of conferred LoA between Highway and Suburbs: the Suburbs scenario marked significantly higher ratings for all three scenes. Additionally, both scenarios noted significant changes in their ratings between different scenes (∗ = *p* < 0.05, ∗∗ = *p* < 0.01, ∗∗∗ = *p* < 0.001).

Here as well we can see the similar scenario trends as in Section 4.3 - participants conferred consistently lower LoA to the AV during the Highway scenario, whereas during the Suburbs scenario their ratings dropped in the unexpected Scene 2, but then remained stable in Scene 3, as illustrated in Figure 12.

With this, we can confirm the hypothesis from RQ 2.2, as we have shown that the scenario and driving environment had a clear impact on the conferred Level of Autonomy.

### 4.5 Exploratory demographics analysis

As a complement to the full statistical analysis on the survey data, we performed an exploratory analysis on participants’ demographic data, with the aim of investigating any correlations between the demographic data, the results of the DBQ and AV-NARS questionnaires, and the participant ratings of the Levels of Autonomy (LoA) and Trust in the AV.

To rule out any confounding effect, we first checked how participants’ age, gender, and driving experience correlated with the four metrics above, and then performed the subsequent analysis controlling for them.

Age was significantly (p<0.05) correlated with all four metrics, specifically a weak-negative correlation with the DBQ (rho(206)=−0.215,p=0.002) , very weak-negative with the AV-NARS (rho(206)=−0.138,p=0.047) , and a very weak-positive with both the LoA rating (rho(206)=0.175,p=0.012) and the Trust rating (rho(206)=0.158,p=0.023) .

Gender had only a weak-negative correlation with the AV-NARS score (rho(206)=−0.264,p<0.001) and a weak-positive with the Trust rating (rho(206)=0.205,p=0.003) .

From the driving experience metrics, the years of being an active driver had a weak-positive correlation with the DBQ score (rho(206)=0.220,p=0.001) , and a very weak-positive with the AV-NARS score (rho(206)=0.167,p=0.016) . The inverse was noted for the other two metrics, with a very weak-negative correlation with both the LoA (rho(206)=−0.148,p=0.034) and Trust (rho(206)=−0.151,p=0.031) ratings. The frequency of driving had only a very weak-negative correlation with the DBQ score (rho(206)=−0.181,p=0.009) .

We then calculated the Spearman rho Correlation Coefficient for the DBQ and AV-NARS scores and the average LoA and Trust ratings, while controlling for the four confounding factors of age, gender, and people’s driving experience (their years of being an active driver, and the frequency of driving).

We found a very weak-negative correlation between participants DBQ and AV-NARS scores (rho(206)=−0.148,p=0.036) , and a very weak-positive correlation between their DBQ score and their Trust rating ( rho (206) = 0.170, p = 0.016), but no correlation between their DBQ and their LoA (rho(206)=0.036,p=0.614) . In other words, participants’ driving style (as it pertained to the errors they commit while driving and their risk-taking behaviour) was only slightly correlated with how they feel towards AVs and how much they’d trust them, but it didn’t affect at all the level of autonomy they’d confer to them.

We did however note a moderate-negative correlation between the AV-NARS scores and the average LoA ratings (rho(206)=−0.325,p<0.001) , as well as a moderate-negative correlation between the AV-NARS score and the Trust ratings (rho(206)=−0.540,p<0.001) . This was in line with the findings from our earlier participatory design study (Tanevska et al., 2023), which also showed that the less negative attitudes towards AVs users may have, the higher their trust in the AV and the level of autonomy they’d be comfortable with the AV possessing.

**FIGURE 13:**
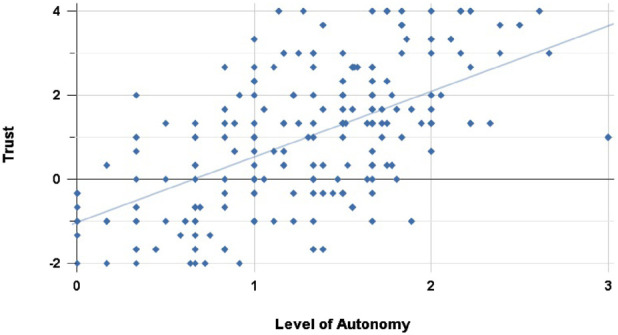
Correlation of Trust and LoA ratings in the Highway scenario: we noted a moderate-strong positive correlation between participants’ Trust and LoA ratings (rho(206)=0.570,p<0.001) .

**FIGURE 14:**
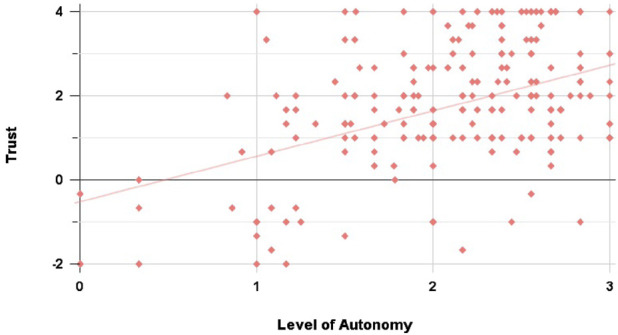
Correlation of Trust and LoA ratings in the Suburbs scenario: we noted a weak-moderate positive correlation between participants’ Trust and LoA ratings (rho(206)=0.375,p<0.001) .

We then additionally looked into the correlations between the average LoA and Trust scores, as well as the separate Highway and Suburbs ratings of the same. We found a general moderate-positive correlation between the Trust and LoA ratings (rho(206)=0.520,p<0.001) , both for the average ratings across the entire study (averaged through both the Highway and Suburbs scenarios), and for the individual scenario ratings (the pairs of Trust/LoA for Highway and Suburbs), with the correlation of LoA and Trust ratings in the Highway scenario bordering on strong-positive (rho(206)=0.570,p<0.001) , and the one in the Suburbs scenario a weak-positive (rho(206)=0.375,p<0.001) . Figures 13, 14 show the individual scenario correlations for Highway and Suburbs respectively.

As the last analysis, we looked at the Trust and LoA ratings individually and compared them across the two scenarios. We found a strong-positive correlation between the Trust ratings in Highway and Suburbs (rho(206)=0.616,p<0.001) , but only a weak-positive correlation between the same ratings for the LoA (rho(206)=0.342,p<0.001) . This implies that while participants’ Trust and Level of Autonomy ratings tended to be correlated, their trust towards AVs seemed to be more intrinsic and stable across evaluations, whereas the Level of Autonomy depended also strongly on the scenario, which was validated also by our confirmatory analysis from Sections 4.1–4.4.

## 5 Discussion

In this paper we presented the findings of our large-scale online study, researching the characteristics that an AV should have in order to be perceived as trustworthy. Specifically, we were interested in investigating how different levels of designed transparency on the AV’s side can influence the user’s comfort and trust in the AV, as well as the maximum level of autonomy they would be comfortable conferring to the AV. The key findings are summarized in Table 4, with the four research questions mapped to their outcomes.

In broad terms, we discovered that the type of environment (Highway vs Suburbs) in which the users were interacting with the AV tended to be the most important factor when selecting a level of autonomy, with the effect sizes ranging from large to very large. The conferred LoA was additionally notably affected by any high-risk or unexpected moments that occurred in the scenarios - the unsafe merging of the second car on the highway, or the unexpected decrease of speed in the empty suburban street. Trust and comfort, on the other hand, were influenced both by the interaction scenario as well as the designed transparency of the AV’s interface (High Information vs Low Information), with the effect sizes being smaller and ranging between small and large (see Table 4 for a breakdown of the effect sizes per analysis).

The overarching goal of our research is to understand how we can build more trustworthy AVs, aligned with the existing guidelines Ethical and Trustworthy AI, such as the ALTAI set (Ala-Pietilä et al., 2020). Transparency is one of the key factors in building trustworthy human-agent interactions, and we know that when a robot’s or agent’s perceived intentions and actual capabilities are unaligned, this can be detrimental to a successful interaction with the person. This makes it vitally important for the robot or agent to be transparent about its abilities, providing information about its inner workings in a way that the human interaction partner can infer how and why the agent behaves the way it does.

Our designed Transparency levels, albeit simple, proved functional, as we saw an evident effect of the High Information interface (where the AV was fully transparent about all of the information it is processing) on the users’ Trust, Comfort, and to a lesser extent, conferred Autonomy as well.

Similarly, while we recognize that the highway/suburb scenario dichotomy posed certain scope limitation as it could not encapsulate all high-risk contexts, we still observed notable different patterns in the user behaviour and their metrics through the two scenarios.

In the Highway scenario, which could be described as the objectively more high-risk one (due to the potential outcome of the risky moment), all metrics noted a linear decrease through the scenes. Despite the AV providing a successful resolution to the high-risk moment, the situation itself was deemed to be stressful and impacted the trust participants had in the AV. This evaluation was further confirmed by some of the free-text answers users could provide to the “Trust” question, such as “I don’t like things where I do not have control.“, “Reliable but not enough to cross over all those lanes of traffic for me. Too many risky driver behaviour”, or “My anxiousness would be about what could influence the car from outside and not the car itself.”

On the other hand, the Suburbs scenario had a notably different pattern in the relevant metrics throughout the different scenes, with all of them experiencing a drop during the second scene of the unexpected moment (more so in the Low Information condition), but then either recovering or remaining stable during the resolution. These findings, coupled with the correlation results from 4.5, illustrate how the development of trust in AVs is a complex, multi-faceted process. The driving scenario seemed to be a key component when conferring autonomy to the AV, but the AV’s transparency also played an important role in the trust - we could see that even if the users felt uncomfortable with a higher level of autonomy due to a risky situation, their trust remained a bit more stable.

An important note - in this research, we did not present and analyze the free-text responses of the participants mentioned above, as only a handful of participants (out of 206) gave some, resulting in only 14 entries (with some participants giving feedback in all scenes). The full set of the 14 responses can be seen in an early workshop preprint containing some of our preliminary analysis (Tanevska et al., 2025). However, we believe this data is still a valuable source of user feedback on the inherent autonomy-trust dynamics, which can be used in the next iteration of our study. In our next steps, we plan to analyze how the valence of the free-text responses (more positive or negative) correlates with the other metrics in the study (the LoA selected by the participant, their comfort and trust scores, etc.). These findings can then inform the design of a follow-up participatory workshop.

Looking at these findings from a design implications and policy-making perspective, we believe that increasing (and arguing for enforcing) transparency seems to be beneficial for trust. However, future work ought consider any possible ramifications for the users’ cognitive load that might influence the overall driver performance, especially with regards to their ability to (re-)engage in driving. Designers, and regulatory bodies, might consider the need for context-sensitive autonomy frameworks which recommend higher versus lower levels of autonomy according to the driving context. This was also in line with some of the group discussions during our earlier participatory design workshop (Tanevska et al., 2023), where some of the participants mentioned that an interface where the level of information could be modulated would be the ideal solution.

Another point for discussion, which however requires more dedicated exploration than we can provide here, is the extent to which user trust can and ought be prioritized, alongside the proper consideration of objective safety risk. For example,; our results point to increased AV trust in suburban (slower) versus highway (faster) driving conditions. If this is driven by perceived risk associated with speed, could this point to users’ having less perception of risks associated with, e.g., pedestrians and cyclists? Future work could further investigate the average road users’ ability to assess contextual driving risk, if users’ AV-trust is to inform, via regulation or via user control, autonomy level of AV operation.

We are also cognizant of the key limitations of conducting an online study to investigate these types of interactions with AVs, compared to real-world or high-fidelity simulator environments. One notable concern of course is the ecological validity and generalisability of the findings. The static stimuli used in our research (the images and narrative descriptions of the scenes) do not fully capture the sensory, spatial, and temporal dynamics of actual AV interactions. As such, one obvious consequence is that we might have hypothetical bias in the user responses, with the participants imagining how they would behave, rather than demonstrating their actual behavior under realistic conditions. Another risk with the approach is in the potential oversimplified risk perception and behavioral responses from participants. Finally, online samples may not represent the broader population in terms of driving experience, technological familiarity, or cultural context, introducing online study bias. The lack of physical presence in a vehicle also eliminates embodied cues such as motion feedback, peripheral awareness, or stress responses, which are critical in AV-related decision-making.

However, despite its limitations, we argue that conducting an online study offered us several valuable advantages, particularly in the early stages of trustworthy AV research. We were able to quickly and cost-effectively gather data from a diverse and geographically dispersed participant pool, thus capturing a wider range of attitudes and perceptions toward autonomous vehicles. Moreover we could test multiple scenarios and conditions, which would have been logistically and financially impractical to implement in real-world or simulator settings.

In addition to the practical benefits of using an online study for exploratory research and obtaining early user feedback, we see also a deeper systemic advantage to imaginative studies for studying future technologies. In this point, we are very much aligned with (Rahwan et al., 2025), who argue for using experimental methods to simulate future technologies, and collect quantitative measures of the attitudes and behaviors of participants. Predicting the social and behavioral impact of future technologies in this way allows us to guide their development and regulation before these impacts get entrenched. In conclusion, while online human-AV studies may lack the full ecological validity, we believe they can still be useful for capturing high-level cognitive responses—such as risk perception, user comfort, trust, and autonomy preferences—which as metrics are foundational for informing future design implications or policy-making action points.

With this we conclude the second iteration of a longer, user-centered research aiming to create more trustworthy AVs. We believe we contributed towards bridging the gap between building trustworthy and ethical AVs, and proactively involving the final users in a participatory manner, with the final goal of achieving a more natural, trustworthy, and long-term interaction between humans and artificial agents.

## Data Availability

The datasets presented in this study can be found in online repositories. The names of the repository/repositories and accession number(s) can be found below: https://doi.org/10.5281/zenodo.15648215.

## Footnotes

1 Images generated from https://openai.com/dall-e-2

2 Legal sex as reported on their current documents. The self-reported gender ratio instead was 104:101:1 (F:M:X).

3
https://www.prolific.com/

4
https://www.prolific.com/

5
https://doit.medfarm.uu.se/bin/kurt3/page/about-kurt

6 More information on the free-text answers is provided in the Discussion section.

7 The jamovi project (2025). jamovi (Version 2.6) [Computer Software]. Retrieved from https://www.jamovi.org

## References

[B1] Ala-Pietilä P. Bonnet Y. Bergmann U. Bielikova M. Bonefeld-Dahl C. Bauer W. (2020). The assessment list for trustworthy artificial intelligence (ALTAI) Brussels, Belgium: European Commission. Available online at: https://digital-strategy.ec.europa.eu/en/library/assessment-list-trustworthy-artificial-intelligence-altai-self-assessment .

[B2] Barajas N. C. Rouchitsas A. Broo D. G. (2025). “Examining human–robot interactions: design guidelines for trust and acceptance,” in Human-technology interaction: interdisciplinary approaches and perspectives (Springer), 117–137.

[B3] Bartneck C. Kulić D. Croft E. Zoghbi S. (2009). Measurement instruments for the anthropomorphism, animacy, likeability, perceived intelligence, and perceived safety of robots. Int. J. Soc. robotics 1, 71–81. 10.1007/s12369-008-0001-3

[B4] Beydoun G. Low G. Mouratidis H. Henderson-Sellers B. (2009). A security-aware metamodel for multi-agent systems (MAS). Inf. Softw. Technol. 51, 832–845. 10.1016/j.infsof.2008.05.003

[B5] Calvo-Barajas N. Akkuzu A. Castellano G. (2024). Balancing human likeness in social robots: impact on children’s lexical alignment and self-disclosure for trust assessment. ACM Trans. Human-Robot Interact. 13, 1–27. 10.1145/3659062

[B6] Christ P. F. Lachner F. Hösl A. Menze B. Diepold K. Butz A. (2016). “Human-drone-interaction: a case study to investigate the relation between autonomy and user experience,” in Computer Vision–ECCV 2016 workshops: amsterdam, the Netherlands, October 8-10 and 15-16, 2016, proceedings, part II 14 (Springer), 238–253.

[B7] Detjen H. Faltaous S. Pfleging B. Geisler S. Schneegass S. (2021). How to increase automated vehicles’ acceptance through in-vehicle interaction design: a review. Int. J. Human–Computer Interact. 37, 308–330. 10.1080/10447318.2020.1860517

[B8] Ferber J. Weiss G. (1999). Multi-agent systems: an introduction to distributed artificial intelligence. Reading: Addison-Wesley, 1.

[B9] Gao Y. Sibirtseva E. Castellano G. Kragic D. (2019). “Fast adaptation with meta-reinforcement learning for trust modelling in human-robot interaction,” in 2019 IEEE/RSJ International Conference on Intelligent Robots and Systems (IROS), Macau, China, 03-08 November 2019 (IEEE), 305–312.

[B10] Ha T. Kim S. Seo D. Lee S. (2020). Effects of explanation types and perceived risk on trust in autonomous vehicles. Transp. Res. part F traffic Psychol. Behav. 73, 271–280. 10.1016/j.trf.2020.06.021

[B11] Hancock P. A. Kessler T. T. Kaplan A. D. Brill J. C. Szalma J. L. (2021). Evolving trust in robots: specification through sequential and comparative meta-analyses. Hum. factors 63, 1196–1229. 10.1177/0018720820922080 32519902

[B12] Hou M. Mahadevan K. Somanath S. Sharlin E. Oehlberg L. (2020). “Autonomous vehicle-cyclist interaction: peril and promise,” in Proceedings of the 2020 CHI conference on human factors in computing systems, 1–12.

[B14] Khavas Z. R. Ahmadzadeh S. R. Robinette P. (2020). “Modeling trust in human-robot interaction: a survey,” in Social Robotics: 12th International Conference, ICSR 2020, Golden, CO, USA, November 14–18, 2020 (Springer), 529–541.

[B15] Koo J. Kwac J. Ju W. Steinert M. Leifer L. Nass C. (2015). Why did my car just do that? Explaining semi-autonomous driving actions to improve driver understanding, trust, and performance. Int. J. Interact. Des. Manuf. (IJIDeM) 9, 269–275. 10.1007/s12008-014-0227-2

[B16] Lee H. Samuel S. (2024). Classification of user preference for self-driving mode and behaviors of autonomous vehicle. IEEE Trans. Intelligent Veh., 1–12. 10.1109/tiv.2024.3385789

[B17] Lee Y. M. Madigan R. Giles O. Garach-Morcillo L. Markkula G. Fox C. (2021). Road users rarely use explicit communication when interacting in today’s traffic: implications for automated vehicles. Cognition, Technol. and Work 23, 367–380. 10.1007/s10111-020-00635-y

[B18] Mackay A. Fortes I. Santos C. Machado D. Barbosa P. Boas V. V. (2020). “The impact of autonomous vehicles’ active feedback on trust,” in Advances in Safety Management and Human Factors: Proceedings of the AHFE 2019 International Conference on Safety Management and Human Factors, Washington DC, USA, July 24-28, 2019 (Springer), 342–352.

[B19] Malik S. Khan M. A. El-Sayed H. (2021). Collaborative autonomous Driving—A survey of solution approaches and future challenges. Sensors 21, 3783. 10.3390/s21113783 34072603 PMC8198430

[B20] Morra L. Lamberti F. Pratticó F. G. La Rosa S. Montuschi P. (2019). Building trust in autonomous vehicles: role of virtual reality driving simulators in hmi design. IEEE Trans. Veh. Technol. 68, 9438–9450. 10.1109/tvt.2019.2933601

[B21] Müller L. Risto M. Emmenegger C. (2016). “The social behavior of autonomous vehicles,” in Proceedings of the 2016 ACM international joint conference on pervasive and ubiquitous computing: adjunct, 686–689.

[B22] Nomura T. Kanda T. Suzuki T. Kato K. (2004). “Psychology in human-robot communication: an attempt through investigation of negative attitudes and anxiety toward robots,” in RO-MAN 2004. 13th IEEE international workshop on robot and human interactive communication (IEEE catalog no. 04TH8759) (IEEE), 35–40.

[B23] Okamura K. Yamada S. (2020). “Calibrating trust in human-drone cooperative navigation,” in 2020 29th IEEE international conference on robot and human interactive communication (RO-MAN) (IEEE), 1274–1279.

[B24] Petersen L. Robert L. Yang X. J. Tilbury D. M. (2019). Situational awareness, drivers trust in automated driving systems and secondary task performance. arXiv preprint arXiv:1903.05251.

[B25] Pinto A. Sousa S. Simões A. Santos J. (2022). A trust scale for human-robot interaction: translation, adaptation, and validation of a human computer trust scale. Hum. Behav. Emerg. Technol. 2022, 6437441. 10.1155/2022/6437441

[B26] Raats K. Fors V. Pink S. (2020). Trusting autonomous vehicles: an interdisciplinary approach. Transp. Res. Interdiscip. Perspect. 7, 100201. 10.1016/j.trip.2020.100201

[B27] Rahwan I. Shariff A. Bonnefon J.-F. (2025). The science fiction science method. Nature 644, 51–58. 10.1038/s41586-025-09194-6 40770437

[B28] Reason J. Manstead A. Stradling S. Baxter J. Campbell K. (1990). Errors and violations on the roads: a real distinction? Ergonomics 33, 1315–1332. 10.1080/00140139008925335 20073122

[B29] Rettenmaier M. Bengler K. (2021). The matter of how and when: comparing explicit and implicit communication strategies of automated vehicles in bottleneck scenarios. IEEE Open J. Intelligent Transp. Syst. 2, 282–293. 10.1109/ojits.2021.3107678

[B13] SAE International Recommended Practice (2018). Taxonomy and definitions for terms related to driving automation systems for on-road motor vehicles. SAE Standard J3016_202104. 10.4271/J3016_201806

[B30] Schwarting W. Pierson A. Alonso-Mora J. Karaman S. Rus D. (2019). Social behavior for autonomous vehicles. Proc. Natl. Acad. Sci. 116, 24972–24978. 10.1073/pnas.1820676116 31757853 PMC6911195

[B31] Shahrdar S. Park C. Nojoumian M. (2019). “Human trust measurement using an immersive virtual reality autonomous vehicle simulator,” in Proceedings of the 2019 AAAI/ACM conference on AI, ethics, and Society, 515–520.

[B32] Sheng S. Pakdamanian E. Han K. Kim B. Tiwari P. Kim I. (2019). “A case study of trust on autonomous driving,” in 2019 IEEE intelligent transportation systems conference (ITSC) (IEEE), 4368–4373.

[B33] Sripada A. Bazilinskyy P. de Winter J. (2021). Automated vehicles that communicate implicitly: examining the use of lateral position within the Lane. Ergonomics 64, 1416–1428. 10.1080/00140139.2021.1925353 33950791

[B34] Tanevska A. Ghosh A. Winkle K. Castellano G. Soudjani S. (2023). “Communicating awareness: designing a framework for cognitive human-agent interaction for autonomous vehicles,” in Cars as social agents (CarSA): a perspective shift in human-vehicle interaction.

[B35] Tanevska A. Winkle K. Castellano G. (2025). “ i don’t like things where i do not have control”: participants’ experience of trustworthy interaction with autonomous vehicles. arXiv preprint arXiv:2503.15522.

[B36] Winkle K. Senft E. Lemaignan S. (2021). Leador: a method for end-to-end participatory design of autonomous social robots. Front. Robotics AI 8, 704119. 10.3389/frobt.2021.704119 34926589 PMC8678512

